# Large-sized grafts versus standard-sized grafts combined with anterolateral ligament reconstruction in ACL-deficient knees: a randomized controlled trial

**DOI:** 10.1186/s43019-026-00321-9

**Published:** 2026-05-08

**Authors:** Mohamed Ali, Omar Abdelkarim, Yasser Soroor, Wael Salama, Moustafa Elsayed, Hossam El-Azab

**Affiliations:** Department of Orthopedics, Sohag University, Sohag, Egypt

**Keywords:** ACL reconstruction (ACLR), Anterior cruciate ligament (ACL), Anterolateral ligament (ALL), Graft failure, Pivot shift, Rotational knee stability

## Abstract

**Background:**

Anterior cruciate ligament (ACL) injuries are highly prevalent among athletes and continue to pose challenges owing to persistent instability and variable return-to-sport outcomes following reconstruction. Anterolateral ligament (ALL) reconstruction and has been introduced to improve outcomes. Increasing graft diameter was described to enhance biomechanical properties. This study hypothesis was, that ACL reconstruction (ACL-R) combined with ALL reconstruction is superior to a large-sized graft ACL-R.

**Purpose:**

To compare outcomes of large-sized (six-strand) hamstring grafts with those of standard-sized (four-strand) grafts combined with anterolateral ligament (ALL) reconstruction in ACL-deficient knees.

**Methods:**

A total of 82 patients (18–45 years) undergoing ACL reconstruction were randomized to either a large (six-strand) hamstring graft group (group A, *n* = 41) or a standard-sized (four-strand) graft plus ALL reconstruction group (group B, *n* = 41). Primary outcomes were knee stability (pivot-shift and Lachman tests) and functional scores [Lysholm and International Knee Documentation Committee (IKDC) scores]. Secondary measures included pain scores, return to sport, and complication rates, with follow-up at 24 months.

**Results:**

The mean diameter of the large-sized graft was 9.5 ± 2.5 mm, while the mean diameter of the standard-sized graft was 8.0 ± 2.0 mm. Both groups demonstrated significant gains in stability and function. Lysholm scores improved from 51 to 94 in group A and from 56 to 98 in group B with no significant difference between both groups (*p* = 0.418), while IKDC scores rose from 37 to 88 and from 37 to 91, respectively and it was significantly higher in group B (*p* = 0.036). Negative pivot-shift was observed in 87.8% of group A and 90.2% of group B with no significant intergroup difference (*p* = 0.841). Return-to-sport at 12 months was 93.5% and 96.1%, respectively with no significant difference (*p* = 1.00). Graft rupture occurred in 4.8% of group A and 2.4% of group B. Overall complications were low and statistically comparable (*p* = 1.00).

**Conclusions:**

Both large hamstring grafts and standard grafts augmented with ALL reconstruction provided significant functional and stability improvements, with no major differences between techniques.

## Introduction

Anterior cruciate ligament (ACL) rupture is among the most common knee injuries in sports and high-demand activities, often resulting in long-term morbidity and prolonged time away from competition. Reconstruction of the ACL (ACLR) is considered the gold standard for restoring stability and function and aims to return individuals to their preinjury activity levels. Despite advancements in surgical techniques and rehabilitation protocols, outcomes remain variable, with residual instability and graft failure still being reported [1].

Anterolateral knee instability has been identified as an important factor influencing postoperative outcomes. Injury to the anterolateral ligament (ALL) and surrounding capsular structures frequently accompanies ACL injuries. These structures act as secondary stabilizers against internal tibial rotation and contribute to the pivot-shift phenomenon. If left untreated, anterolateral deficiency may overload the ACL graft, increase the risk of meniscal injury, and predispose to graft failure [2–4]. Clinically, this condition can be detected through pivot-shift and Lachman tests, while high-resolution magnetic resonance imaging (MRI) may assist in confirming ALL injury [3]. To address this problem, adjunctive procedures such as lateral extra-articular tenodesis (LET) and ALL reconstruction (ALLR) have been introduced to enhance rotational control, lower revision rates, and improve return-to-sport outcomes, particularly in young and high-risk patients [5].

Recent high-level evidence suggests that adjunctive anterolateral procedures may be particularly indicated in patients with high-grade pivot shift (grade II–III), as this finding reflects significant rotatory instability and is associated with an increased risk of graft failure [2–6]. Accordingly, patients with pivot-shift grade II–III represent a population in whom the role of adjunctive ALL reconstruction should be further evaluated.

Another determinant of ACLR outcomes is graft diameter. Several studies have shown that hamstring autografts smaller than 8 mm are associated with higher revision rates and mechanical insufficiency [7, 8]. Nevertheless, graft size alone does not fully determine surgical success, as tunnel placement, graft type, and postoperative rehabilitation also play significant roles [9]. While larger grafts are generally preferred for their superior biomechanical properties, the definition of an “optimal” graft size remains controversial, as individual factors such as age, activity level, and knee morphology influence graft sizing [10].

Importantly, most available literature has focused on graft size in isolated ACLR, leaving uncertainty about outcomes when ACLR is combined with ALLR. It also remains unclear whether the additional rotational stability provided by ALLR can adequately compensate for the limitations of smaller grafts [7–10].

Based on this background, the present randomized trial was designed to compare two surgical approaches: ACLR using large hamstring grafts and ACLR with standard-sized grafts supplemented by ALL reconstruction. The primary objective was to evaluate differences in knee stability, functional outcomes, and graft survival. To the best of our knowledge, no prior study has directly compared these two approaches, making this investigation an important contribution toward evidence-based recommendations for graft selection and the role of adjunctive ALLR in managing ACL-deficient knees with rotatory instability.

### Research question

Do large-sized ACL grafts alone provide comparable or superior outcomes compared with standard-sized ACL grafts combined with ALL reconstruction as regard knee stability, functional outcomes, complication rates (e.g., infection, stiffness, graft failure, reoperation), graft survival, and long-term durability in ACL-deficient knees? By comparing the results of both surgical techniques, we can determine if it is mandatory to do and when to do ALL reconstruction in cases of ACL injuries according to graft size.

## Patients and methods

This prospective, randomized controlled clinical trial enrolled 82 patients between 18 and 45 years of age who presented with ACL rupture confirmed clinically and by MRI. All participants exhibited signs of anterolateral rotatory instability (ALRI), defined as pivot-shift grade II or III, and had a body mass index (BMI) below 35. We used six-strand hamstring autograft (triple semitendinosus and gracilis) as a large-sized graft and four-strand hamstring autograft (triple semitendinosus and single gracilis) as a standard-sized graft. Recruitment took place from January 2022 to December 2024 at the Orthopedic Sports Medicine Clinic of our institution. Ethical approval was obtained from the institutional scientific and ethical committee, and all participants provided informed consent prior to enrollment.

### Sample size calculation

An a priori sample size calculation was conducted using G*Power software (version 3.1.9.4). The calculation was based on previously published studies evaluating clinically meaningful changes in the International Knee Documentation Committee Subjective Knee Form (IKDC-SKF) following anterior cruciate ligament reconstruction. Ohji et al. [11] reported that a minimum sample size of 25 participants was required to detect significant preoperative-to-postoperative changes using a two-tailed test with an effect size of 0.76, an *α* error probability of 0.05, and a statistical power of 0.95. In addition, Kerdtho and Lertwanich [12] demonstrated large responsiveness of the IKDC subjective score after ACL injury, reporting a mean change of approximately 15 points with a standard deviation of about 15 points and a large effect size. Based on these findings, and assuming an *α* level of 0.05 and a power of 0.90, a sample size of 25 participants per group was considered adequate to detect clinically meaningful changes in IKDC scores. This sample size was further selected to enhance the robustness of the analysis and to account for potential loss to follow-up.

Inclusion criteria were ACL tears associated with ALRI (pivot-shift grade II–III) and no prior knee surgery. Exclusion criteria were multiple-ligament knee injuries, malalignment > 3° (varus or valgus), cartilage lesions or osteoarthritis exceeding Outerbridge grade II, neuromuscular disorders, or generalized ligamentous laxity.

To assign the participants, a computer-generated random number table was utilized (allocation ratio 1:1) either to group A: (Large Graft Group) or group B: (Standard Graft + ALL Group). Assignment was done by an independent biostatistician who was not included in patient care or outcomes evaluation. Allocation concealment was achieved by sealed opaque envelopes. Outcome assessors, who performed pivot-shift grading and functional scoring, were blinded to group assignment.

Group A (Large Graft Group): Underwent ACL reconstruction with a six-strand hamstring autograft (triple semitendinosus and gracilis) measuring > 9 mm in diameter, fixed with bioabsorbable interference screws.

Group B (Standard Graft + ALL Group): Underwent ACL reconstruction with a four-strand hamstring autograft (triple semitendinosus and single gracilis, ~8 mm diameter), combined with anatomical ALL reconstruction using the residual gracilis tendon. Fixation was performed with bioabsorbable interference screws.

All operations were performed by a single senior surgeon using an anatomical ACLR technique. Graft diameters were measured intraoperatively using calibrated sizing tubes. Pivot-shift grading followed Jakob’s classification system. [13, 14]

### Surgical technique

For both groups, hamstring autografts were harvested via the standard technique. Tunnels were drilled anatomically, and fixation achieved with bioabsorbable interference screws (Smith & Nephew Endoscopy) [15].

**Fig. 1:**
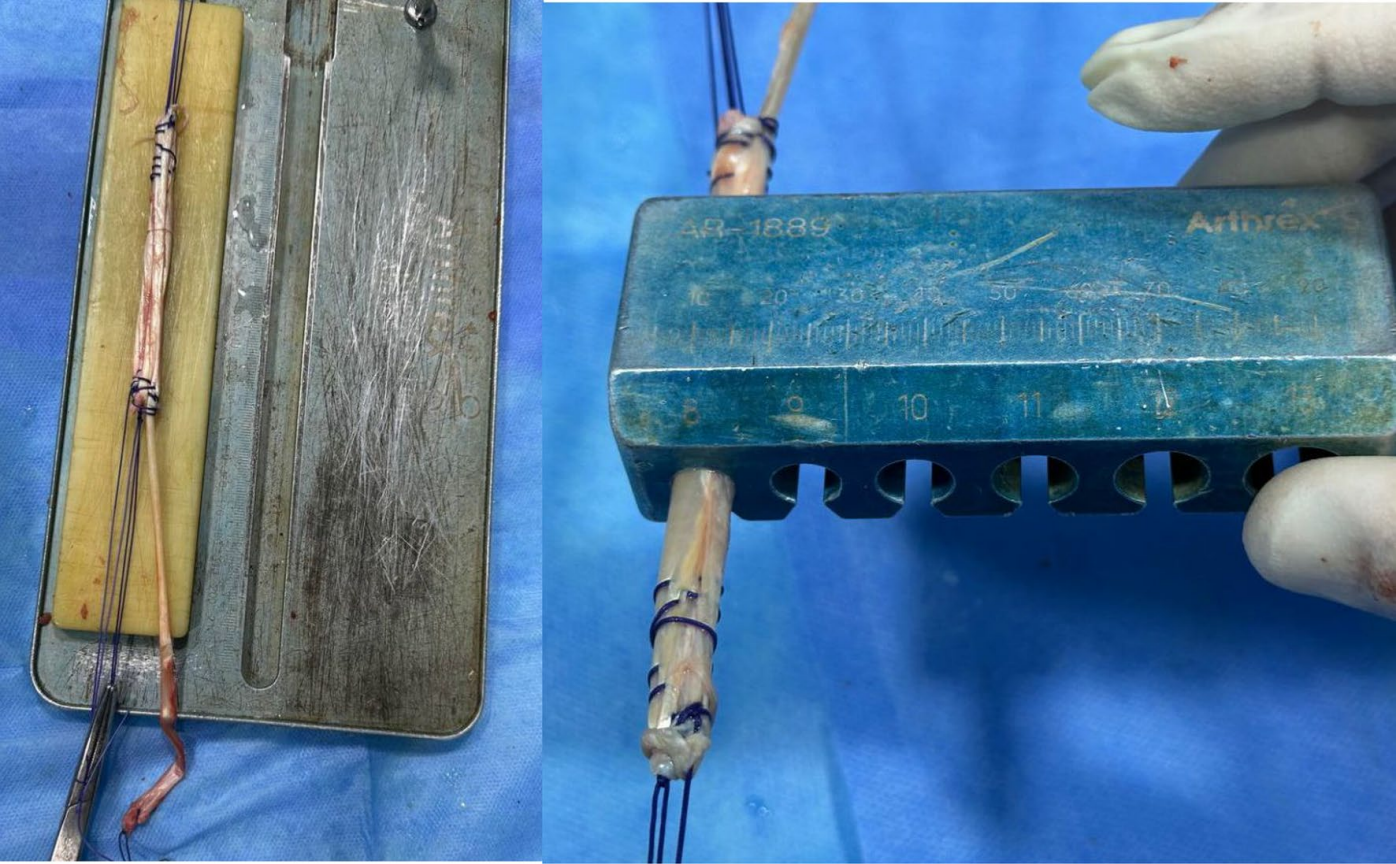
Hamstring autograft preparation (triple semitendinosus and single gracilis; 4 strands; ~8 mm diameter)

**Fig. 2:**
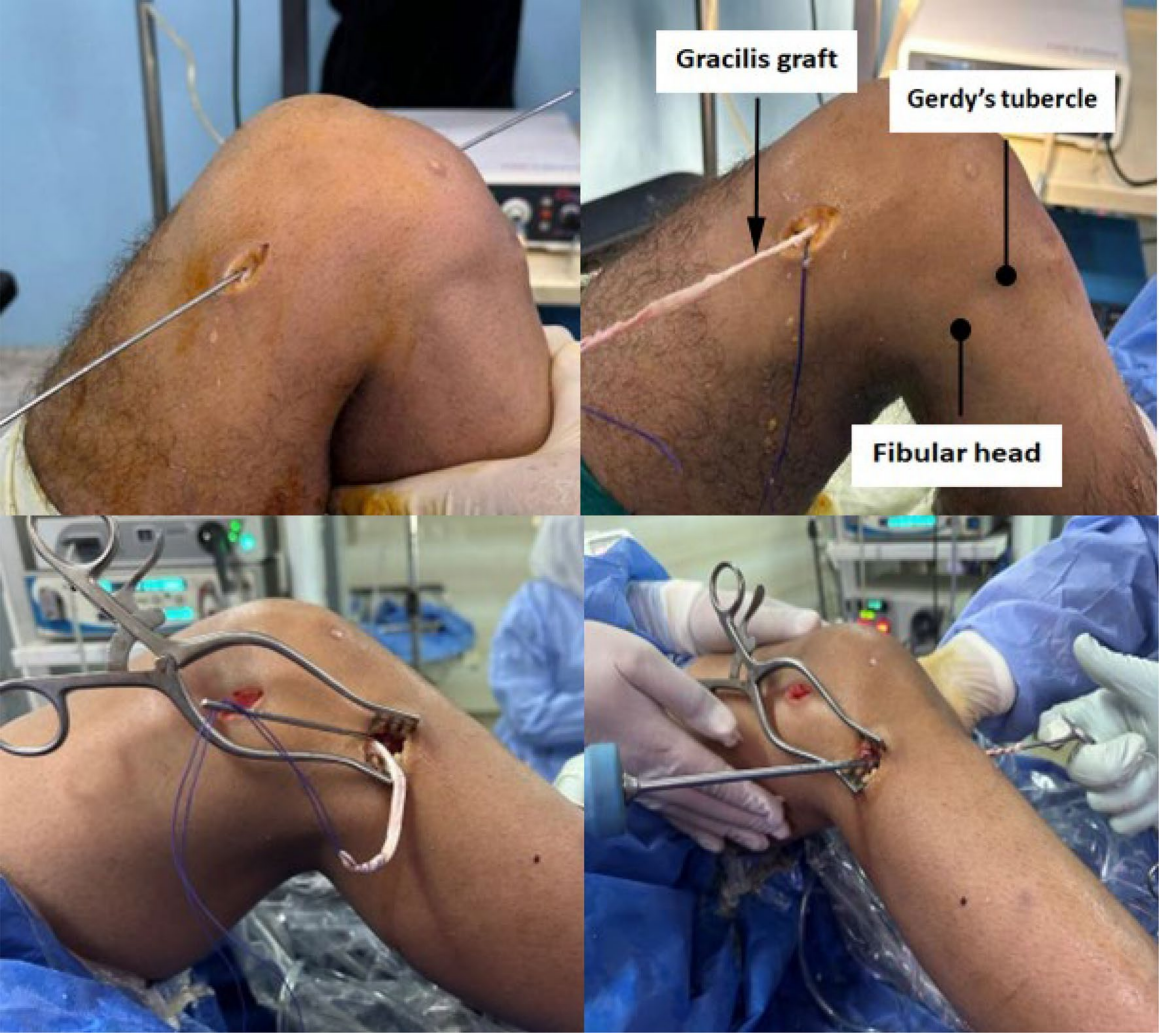
The remaining gracilis graft passed through the subcutaneous and ALL tunnels; fixation at a point halfway between Gerdy’s tubercle and the fibular head with a bioabsorbable interference screw

**Fig. 3:**
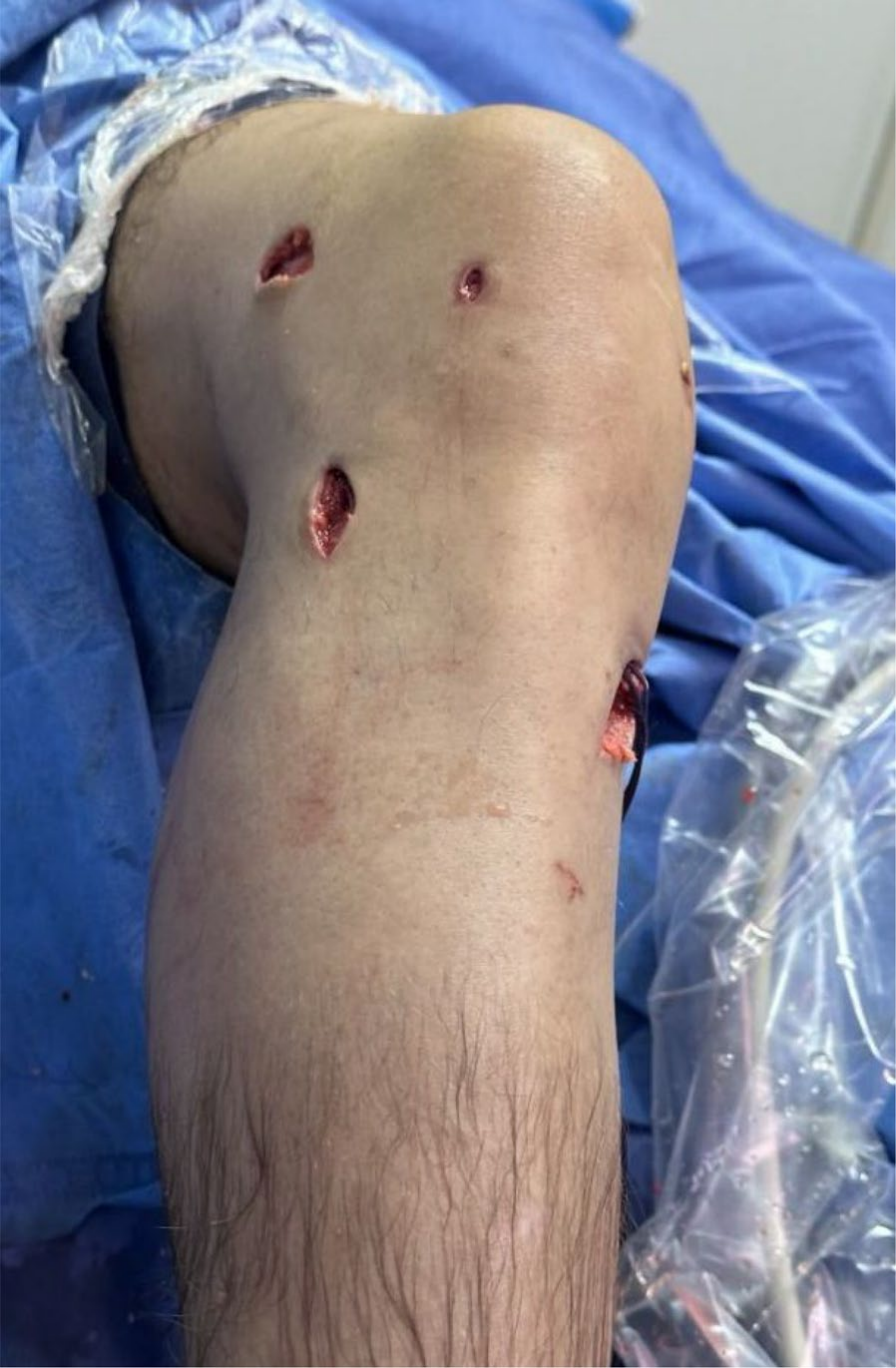
Final intraoperative photo after minimally invasive anatomical ALL reconstruction

Group A: employed a six-strand construct. Group B: Utilized a four-strand construct. The residual gracilis was used for ALL reconstruction. (Fig. 1) Fixation was performed at a point midway between Gerdy’s tubercle and the fibular head, approximately 1.5 cm below the joint line, with the knee in near-full extension and slight valgus (Figs. 2 and 3).

Concomitant meniscal injuries, when present, were addressed in the same surgical setting. Meniscal tears located in the vascular zone were managed with arthroscopic repair, whereas irreparable or degenerative tears were treated with partial meniscectomy.

### Follow-up and outcomes

Patients were followed at 2, 4, and 6 weeks, and at 3, 6, 12, and 24 months, postoperatively.

Primary outcomes: knee stability (pivot-shift and Lachman tests) and functional outcomes were assessed using the validated Arabic version of the Lysholm score and IKDC Subjective Knee Form [16].

Secondary outcomes: pain intensity using visual analogue scale (VAS), return-to-sport at the preinjury level, and complications (infection, stiffness, arthrofibrosis, graft failure, reoperation).

### Rehabilitation protocol

All patients followed the same standardized rehabilitation program:

- 0–6 weeks: weight-bearing as tolerated, continuous passive motion from 0°–90°, swelling control, quadriceps strengthening.
- For patients with meniscal repair: partial weight-bearing was allowed, knee flexion was limited to < 90°, and squatting/twisting were avoided. 6–12 weeks: progressive range of motion (ROM) recovery, strengthening, and proprioceptive training.

With meniscal repair: gradual WB to full by 6 weeks, initiate ROM beyond 90°.

- 3–6 months: flexibility and sport-specific strengthening.

With meniscal repair: delay pivoting/cutting until 4–5 mo.

- 6–9 months: progressive sport drills and functional training.
-  > 9 months: return to sport allowed once functional and clinical criteria were met.

With meniscal repair: RTS usually 9–12 mo depending on healing.

### Statistical analysis

Data analysis was performed using SPSS version 29.0 (IBM, Armonk, NY). Distribution was tested with the Shapiro–Wilk method and histogram inspection. Parametric variables were expressed as mean ± standard deviation and compared using independent Student’s *t*-test. Nonparametric data were summarized as median and interquartile range (IQR) and analyzed with the Mann–Whitney *U* test. Categorical variables were expressed as frequencies and percentages, with chi-square or Fisher’s exact test applied when appropriate. A *p*-value < 0.05 was considered statistically significant.

## Results

**Fig. 4:**
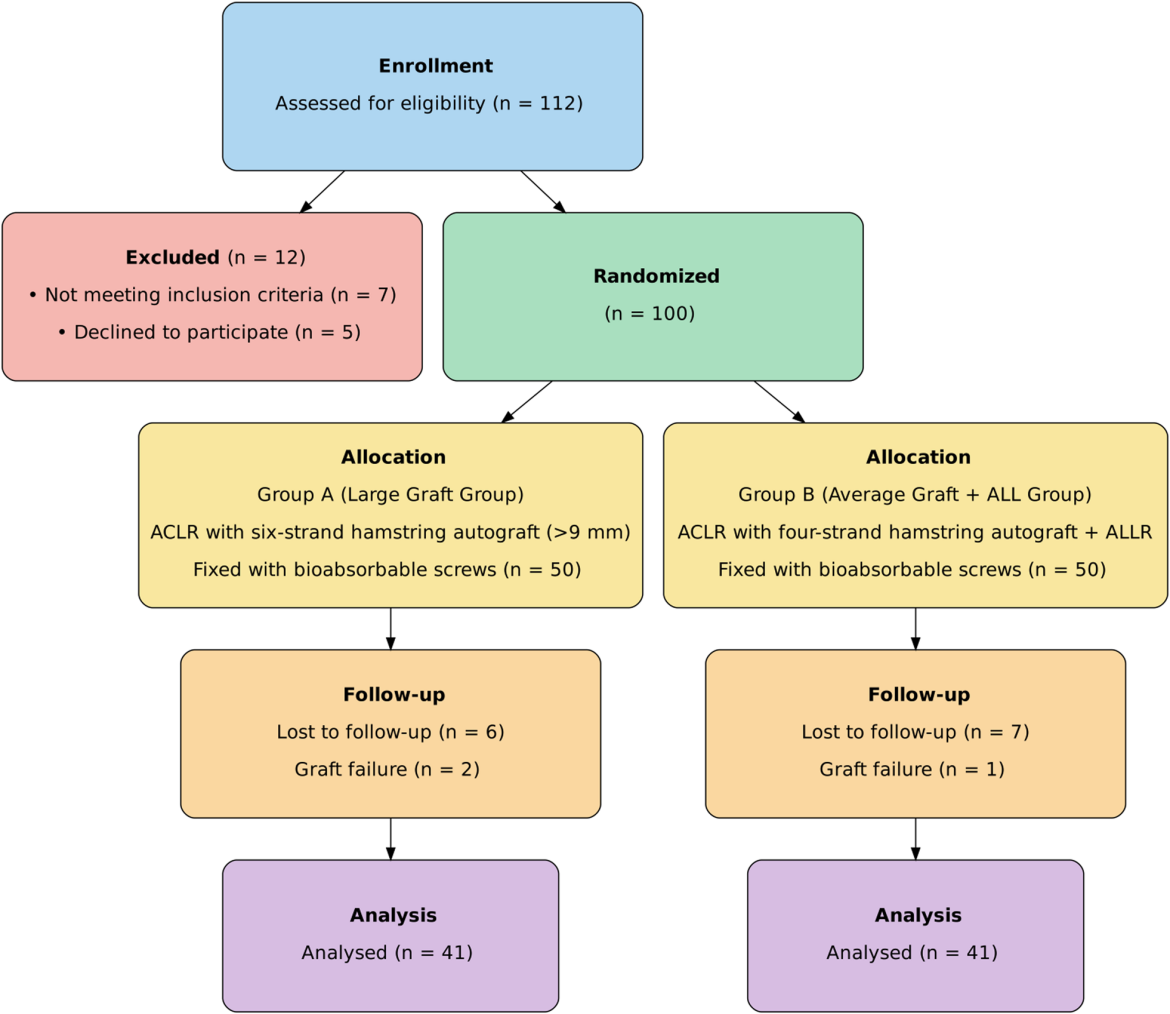
CONSORT Flow Diagram showing patient enrollment, allocation, follow-up, and analysis

Out of 112 screened patients, 12 were excluded (7 did not meet inclusion criteria, 5 declined participation). The remaining 100 patients were randomized equally. During the follow-up period (mean 24 ± 6 months), 6 participants in group A and 7 in group B were lost, while 2 and 1, respectively, experienced graft failure. Consequently, 41 patients per group completed the study and were included in the final analysis (Fig. 4).

Both groups were comparable at baseline, with no significant differences in demographic or clinical variables including age, BMI, sex, side or mechanism of injury, proportion of athletes, or duration from trauma to surgery (*p* > 0.05). Details are shown in (Table 1). The mean diameter of the large-sized graft was 9.5 ± 2.5 mm, while the mean diameter of the standard-sized graft was 8.0 ± 2.0 mm. The mean diameter of residual gracilis used for ALL reconstruction was 6.0 ± 1.5 mm.

**Table 1:** Demographic data of the studied groups

			Group A (*n* = 41)	Group B (*n* = 41)	*p*-value
Age (years)			26.22 ± 5.45	27.02 ± 5.5	0.508
BMI (kg/m^2^)			21.25 ± 1.86	21.48 ± 1.72	0.568
Sex	Male		35 (85.37%)	33 (80.49%)	0.557
	Female		6 (14.63%)	8 (19.51%)	
Side of injury	Right		30 (73.17%)	27 (65.85%)	0.472
	Left		11 (26.83%)	14 (34.15%)	
Mechanism of injury	Sport injury		28 (68.29%)	25 (60.98%)	0.780
	Motor car		6 (14.63%)	7 (17.07%)	
	Other		7 (17.07%)	9 (21.95%)	
Number of athletes	Professional		8 (19.51%)	7 (17.07%)	0.485
	Athletes		23 (56.1%)	19 (46.34%)	
	Nonathletes		10 (24.39%)	15 (36.59%)	
Meniscal injury	Present		26 (63.41%)	27 (65.85%)	0.802
	Managed by	Repair	9 (21.95%)	7 (17.07%)	
		Partial meniscectomy	17 (41.46%)	20 (48.78%)	
	Absent		15 (36.59%)	14 (34.15%)	
Duration from trauma to surgery (months)			7.63 ± 4.25	6.66 ± 4.03	0.289

Data are presented as mean ± SD or frequency (%). BMI, Body mass index

### Pivot-shift test

Preoperatively, most patients demonstrated grade II or III pivot shift. At 24 months, a negative pivot-shift was observed in 36 patients (87.8%) in group A and 37 (90.2%) in group B. Only a small number in each group exhibited grade I–II laxity, with no significant intergroup difference (*p* = 0.841) (Table 2).

**Table 2:** Pivot shift test results in the studied groups

			Group A (*n* = 41)	Group B (*n* = 41)	*P*-value
Pivot shift test	Preoperative	Positive GII	11 (26.8%)	14 (34.15%)	0.471
		Positive GIII	30 (73.2%)	27 (65.85%)	
	Postoperative	Negative	36 (87.8%)	37 (90.2%)	0.841
		Positive GI	3 (7.3%)	3 (7.3%)	
		Positive GII	2 (4.9%)	1 (2.4%)	

Data are presented as frequency (%)

### Lachman test

Both groups showed marked improvement in Lachman grades postoperatively (*p* < 0.001 within groups). At final follow-up, 87.8% of group A and 90.2% of group B tested negative, with no significant difference between groups (*p* = 0.92) (Table 3).

**Table 3:** Lachman test of the studied groups

			Group A (*n* = 41)	Group B (*n* = 41)	*P*-value
Lachman test	Preoperative	Positive GI	3 (7.3%)	4 (9.8%)	0.920
		Positive GII	18 (43.9%)	16 (39.0%)	
		Positive GIII	20 (48.8%)	21 (51.2%)	
	Postoperative	Negative	36 (87.8%)	37 (90.2%)	0.920
		Positive GI	4 (9.8%)	3 (7.3%)	
		Positive GII	1 (2.4%)	1 (2.4%)	

Data are presented as frequency (%)

### Lysholm score

**Fig. 5:**
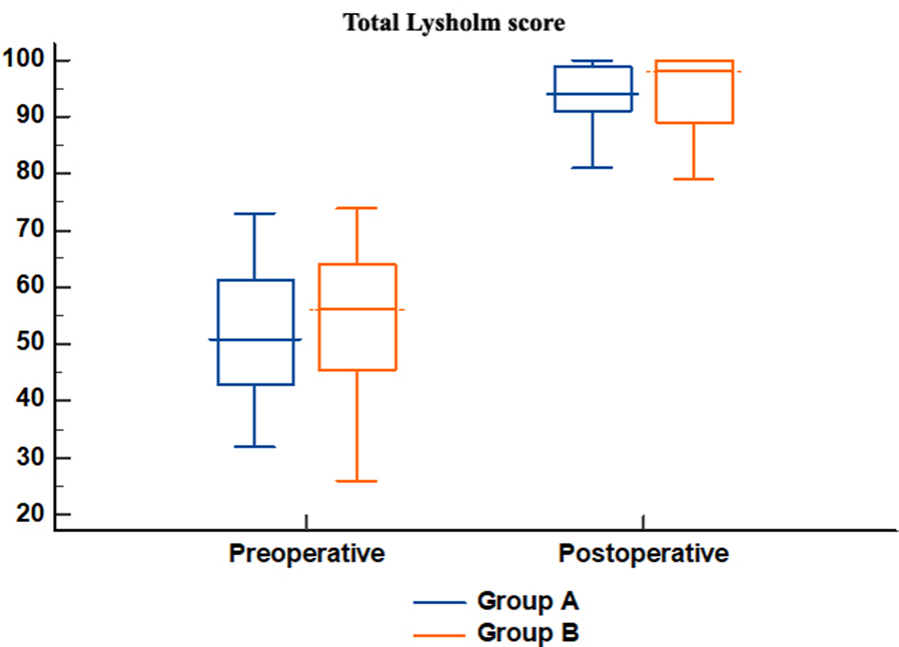
Total Lysholm score of the studied groups

All Lysholm subdomains (limp, support, locking, instability, pain, swelling, stair climbing, and squatting) improved significantly within each group compared with baseline (*p* < 0.05). At 2-year follow-up, the total Lysholm score reached 94 (91–99) in group A and 98 (89–100) in group B, with no significant difference (*p* = 0.418) (Table 4; Fig. 5).

**Table 4:** Lysholm score of the studied groups (preoperative and postoperative (at 2-year follow up))

		Group A (*n* = 41)	Group B (*n* = 41)	*P*-value
Limp	Preoperative	3 (0–3)	3 (0–3)	0.507
	Postoperative	5 (3–5)	5 (5–5)	0.620
	P^#^	< 0.001*	< 0.001*	
Support	Preoperative	5 (2–5)	5 (2–5)	0.484
	Postoperative	5 (5–5)	5 (5–5)	0.646
	P^#^	< 0.001*	0.004*	
Locking	Preoperative	10 (6–10)	10 (6–10)	0.171
	Postoperative	15 (15–15)	15 (15–15)	0.776
	*p*^#^	< 0.001*	< 0.001*	
Instability	Preoperative	10 (5–10)	10 (5–10)	0.573
	Postoperative	25 (25–25)	25 (25–25)	0.596
	*p*^#^	< 0.001*	< 0.001*	
Pain	Preoperative	15 (10–20)	15 (15–20)	0.127
	Postoperative	25 (25–25)	25 (25–25)	0.639
	*p*^#^	< 0.001*	< 0.001*	
Swelling	Preoperative	6 (6–6)	6 (6–6)	0.826
	Postoperative	10 (6–10)	10 (10–10)	0.335
	*p*^#^	< 0.001*	< 0.001*	
Stair-climbing	Preoperative	6 (2–6)	6 (6–6)	0.132
	Postoperative	10 (6–10)	10 (10–10)	0.801
	*p*^#^	< 0.001*	< 0.001*	
Squatting	Preoperative	4 (2–4)	4 (4–4)	0.897
	Postoperative	5 (5–5)	5 (4–5)	0.273
	*p*^#^	< 0.001*	< 0.001*	
Total	Preoperative	51 (43–61)	56 (46–64)	0.162
	Postoperative	94 (91–99)	98 (89–100)	0.418
	*p*^#^	< 0.001*	< 0.001*	

Significance of bold values means *p*-value < 0.05

Values are presented as median (IQR). *p* = comparison between group A and group B. *p*# = comparison pre- versus postoperative within each group. * indicates statistically significant (*p* < 0.05)

### IKDC score

**Fig. 6:**
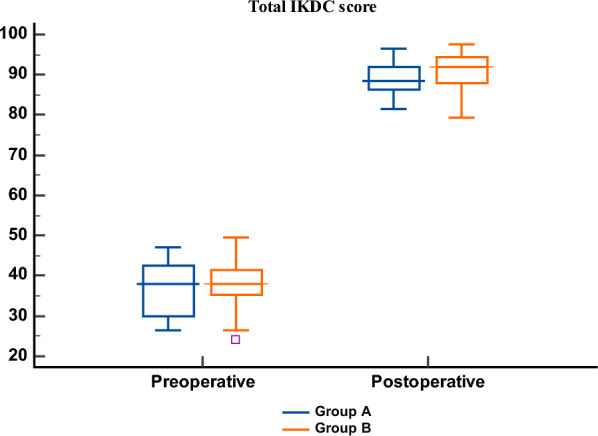
Total IKDC score of the studied groups

Within both groups, IKDC subcomponents and total scores improved significantly (*p* < 0.001). At final follow-up, the total IKDC score was higher in group B (91 [88–94]) compared with group A (88 [86–91]) (*p* = 0.036). Current knee function was also superior in group B (*p* = 0.006) (Table 5; Fig. 6).

**Table 5:** IKDC score of the studied groups (preoperative and postoperative (at 2-year follow up))

		Group A (*n* = 41)	Group B (*n* = 41)	*p*-value
Max painless action	Preoperative	1 (0–1)	1 (0–1)	1
	Postoperative	4 (3–4)	4 (3–4)	0.815
	*p*^#^	< 0.001*	< 0.001*	
Pain frequency	Preoperative	2 (1–3)	2 (1–3)	0.808
	Postoperative	9 (8–9)	9 (8–9)	0.698
	*p*^#^	< 0.001*	< 0.001*	
Pain severity	Preoperative	2 (1–3)	2 (1–3)	0.860
	Postoperative	8 (8–9)	9 (8–9)	0.768
	*p*^#^	< 0.001*	< 0.001*	
Stiffness	Preoperative	2 (2–3)	3 (2–3)	0.399
	Postoperative	4 (4–4)	4 (4–4)	0.580
	*p*^#^	< 0.001*	< 0.001*	
Swelling	Preoperative	1 (1–2)	1 (1–2)	0.587
	Postoperative	4 (3–4)	4 (3–4)	0.815
	*p*^#^	< 0.001*	< 0.001*	
Locking	Preoperative	0 (0–1)	1 (0–1)	0.380
	Postoperative	1 (1–1)	1 (1–1)	0.239
	*p*^#^	< 0.001*	< 0.001*	
Giving way	Preoperative	1 (1–2)	2 (1–2)	0.802
	Postoperative	4 (4–4)	4 (4–4)	0.527
	*p*^#^	< 0.001*	< 0.001*	
Sports activity	Preoperative	1 (1–2)	1 (1–2)	0.751
	Postoperative	4 (3–4)	4 (3–4)	0.117
	*p*^#^	< 0.001*	< 0.001*	
Specific activities	Preoperative	17 (14–19)	18 (16–19)	0.171
	Postoperative	32 (30–33)	32 (31–34)	0.156
	*p*^#^	< 0.001*	< 0.001*	
Pre-injury function	Preoperative	4(3–5)	4(4–5)	0.745
Current knee function	Postoperative	9 (9–10)	10 (9–10)	0.006*
	*p*^#^	< 0.001*	< 0.001*	
Total	Preoperative	37 (29–42)	37 (35–41)	0.216
	Postoperative	88 (86–91)	91 (88–94)	0.036*
	*p*^#^	< 0.001*	< 0.001*	

Significance of bold values means *p*-value < 0.05

Values are presented as median (IQR), *p* = comparison between group A and group B, *p*# = comparison pre- versus postoperative within each group, * indicates statistically significant (*p* < 0.05)

### Pain (VAS)

Mean VAS scores decreased significantly after surgery in both groups. At 24 months, pain levels were minimal (1.0 ± 1.4 in group A versus 1.0 ± 1.6 in group B), with no intergroup difference (*p* = 0.98) (Table 6).

**Table 6:** VAS pain score of the studied groups

Time point	Group A (*n* = 41)	Group B (*n* = 41)	*P*-value
Preoperative	7.2 ± 2.1	7.5 ± 2.3	0.530
6 months postoperative	2.2 ± 1.8	2.5 ± 2.1	0.480
24 months postoperative	1.0 ± 1.4	1.0 ± 1.6	0.980

Data are presented as mean ± standard deviation (SD)

### Return to sport

At 9 months, 45.2% of group A and 50% of group B athletes had returned to sport. By 12 months, return-to-sport rates increased to 93.5% and 96.1%, respectively, with no significant difference (*p* = 1.00) (Table 7).

**Table 7:** Return-to-sport rates among athletes in both groups

Time point	Group A (*n* = 31 athletes)	Group B (*n* = 26 athletes)	*P*-value
9 months postoperative	14/31 (45.2%)	13/26 (50.0%)	0.710
12 months postoperative	29/31 (93.5%)	25/26 (96.1%)	1.000

Data are presented as frequency (%). Denominators represent the number of athletes (31 in group A, 26 in group B)

### Complications

Graft rupture occurred in two patients (4.8%) in group A—one early (with infection at 6 weeks) and one late (20 months, traumatic). Group B had one rupture (2.4%) at 22 months post-trauma. Postoperative infection was documented in two patients (4.8%) in group A and three (7.3%) in group B. The overall complication rate was identical in both groups (9.8%), with no significant differences (*p* = 1.00) (Table 8).

**Table 8:** Postoperative complications among the studied groups

Time point	Group A (*n* = 41)	Group B (*n* = 41)	*P*-value
Graft rupture	2 (4.8%)	1 (2.4%)	1.000
Infection	2 (4.8%)	3 (7.3%)	1.000
Total complications	4 (9.8%)	4 (9.8%)	1.000

Data are presented as frequency (%)

All three cases of graft rupture (3.6% overall) were confirmed clinically and by MRI. Each underwent revision ACL reconstruction using an ipsilateral peroneus longus autograft and was excluded from subsequent follow-up analyses to avoid bias.

No cases of arthrofibrosis, clinically significant stiffness, thromboembolism, hardware issues, or donor-site morbidity were reported.

## Discussion

Anterior cruciate ligament (ACL) injuries remain among the most frequent knee injuries in pivoting sports, accounting for more than half of such cases. The main goals of ACL reconstruction (ACLR) are to restore stability, prevent secondary meniscal or chondral damage, and facilitate return to preinjury performance. The anterolateral ligament (ALL) has been recognized as an important stabilizer against rotational stress, and combining ACLR with ALL reconstruction (ALLR) has been shown to reduce reoperation rates and complications compared with isolated ACLR [17].

Graft characteristics, especially diameter, are also critical for surgical success. Small-diameter hamstring grafts have consistently been linked to increased laxity, worse functional recovery, and higher revision rates [18], whereas larger grafts offer biomechanical advantages by reducing meniscal load and joint stress [19].

In this randomized controlled study, both large hamstring grafts alone and standard-sized grafts combined with ALL reconstruction achieved substantial improvements in stability, pain reduction, and functional outcomes. No significant difference was found between the two groups in terms of graft survival, complication rates, or return-to-sport, although patients who underwent combined ACL–ALL reconstruction demonstrated a trend toward better IKDC (91 versus 88, *p* = 0.036), the mean difference was 3 points. Based on previously published data [12], the reported Minimum Clinically Important Difference (MCID) for the IKDC score is approximately 10 points. Therefore, the observed difference does not appear to reach the MCID threshold, suggesting that although the result is statistically significant, its clinical impact may be limited.

In the present study, graft rupture in group A (large graft) occurred in two patients (4.8%). This aligns with previous reports by Figueroa et al. and Conte et al. [20–28], who demonstrated lower failure rates in ACL reconstruction when using quadrupled hamstring autografts with diameters > 8 mm. Similar conclusions were reached by Dahduli et al. and Yang et al. [21, 22], who confirmed the biomechanical and clinical benefits of larger graft diameters. Park et al. [23] further supported this by demonstrating a failure rate of 5.2% for grafts < 8 mm versus 0% for those > 8 mm.

In group B (standard graft + ALLR), a single rupture (2.4%) was observed at 22 months following trauma. These outcomes are consistent with reports by Conteduca et al. and Laboudie et al. [24, 25], which emphasized that adjunctive ALL reconstruction lowers the risk of graft failure in ACLR. Although graft rupture and reoperation rates were slightly higher in group A compared with group B (4.8% versus 2.4%), this difference was not statistically significant (*p* = 1.0).

Postoperative infection rates were low and comparable between the two groups, with no statistically significant difference (*p* = 1.00). The overall complication rate was identical (9.8% each group), supporting that both strategies are safe.

Both groups exhibited significant improvements in pivot-shift and Lachman grades. In group A, 87.8% achieved a negative pivot shift, which is consistent with Firth et al. and Kurtoğlu et al. [26, 27]. In group B, 90.2% had a negative test at 24 months, mirroring the outcomes reported by Bosco et al. [6] in high-grade rotatory laxity cases treated with combined ACL–ALLR. Lachman test results also improved significantly in both groups, with most patients achieving a negative test at final follow-up. These findings are consistent with Baawa-Ameyaw et al. and Mo et al. [28, 29], who confirmed that adequate graft diameter reduces residual anterior laxity. Although intergroup differences were not significant, group B showed a trend toward improved rotational stability.

Functional outcomes also followed this pattern. Lysholm scores improved significantly in both groups: from 51 (43–61) to 94 (91–99) in group A, and from 56 (46–64) to 98 (89–100) in group B. These results agree with Kurtoğlu et al. [27], who reported inferior outcomes with grafts < 8 mm compared with larger grafts, and with Xu et al. [30], who found significantly higher Lysholm scores in patients receiving grafts > 8.5 mm.

In group B, the marked improvement aligns with Lee et al., de Lima et al. [31, 32], who found superior Lysholm and Tegner outcomes with combined ACL–ALLR. Recent meta-analysis by Han et al. [33], also confirmed improved functional outcomes and reduced graft failure with combined procedures. Furthermore, Helito et al. [34] reported good functional outcomes in ACL–ALL procedures, even in cases with smaller hamstring grafts.

IKDC scores also improved significantly in both groups, from 37 (29–42) to 88 (86–91) in group A, and from 37 (35–41) to 91 (88–94) in group B. These findings are consistent with Kurtoğlu et al. and Xu et al. [27–30], who reported higher IKDC with larger grafts (> 8 mm). Group B’s superior improvement supports evidence from Lee et al. (2018), de Lima et al. (2021), Helito et al., and Han et al. [33, 34], who showed that adjunctive ALLR improves functional outcomes and stability. Subdomain analysis in our cohort showed similar results between groups, but postoperative current knee function and total IKDC were significantly higher in group B. However, the difference did not exceed the MCID making its clinical application limited.

Pain levels declined significantly in both groups, with no difference at 2 years, which corresponds with reports that VAS scores normalize after ACLR regardless of graft size [36–38]. Similarly, Vadalà et al. and de Lima et al. [32–39] concluded that ALLR does not alter pain outcomes but provides benefits in terms of stability and survivorship.

Return-to-sport (RTS) rates exceeded 90% by 12 months in both groups, consistent with prior literature showing favorable RTS after either large graft ACLR (Gudas et al.) [40] or combined ACL–ALLR (Gonnachon et al.) [41]. A recent meta-analysis by Mercurio et al. [42] reinforced these findings by demonstrating higher pooled RTS with combined reconstructions compared with isolated ACLR.

### Strengths and limitations

This study’s strengths are its randomized design, standardized surgical technique, and use of validated outcome measures. Limitations include its single-center nature and the examiner-dependent grading of pivot-shift. Variations in rehabilitation adherence may also have affected outcomes. Nevertheless, despite these limitations, the findings add valuable evidence to guide graft selection and the role of ALL reconstruction in managing rotatory instability, and they may inform the design of future multicenter, long-term comparative trials.

## Conclusions

This randomized trial demonstrated that both strategies—using large hamstring grafts alone or standard-sized grafts combined with ALL reconstruction—achieve comparable improvements in knee stability, functional recovery, and return to sport. ALL reconstruction may be particularly valuable when larger autografts are not available.

## Data Availability

The datasets generated and/or analyzed during the current study are available from the corresponding author on reasonable request.
